# Ureteral polyps protruding from the urethra

**DOI:** 10.1186/s12894-021-00786-8

**Published:** 2021-02-10

**Authors:** Zhi-Wei Sun, Jian-Kang Ge, You Wu, Ye-Qing Huang

**Affiliations:** Department of Urology, Affiliated Hospital of Nantong University, No. 20 Xisi Road, Nantong, China

**Keywords:** Ureteral fibro-epithelial polyp, Protrusion through urethra, Ureteroscopy, Case report

## Abstract

**Background:**

Ureteral fibro-epithelial polyp (UFP) is a rare benign ureteral tumor, and surgical removal of the polyps is still the preferred solution. Although many cases have reported polyps extending to the bladder, our case was the first to report a huge UFP that underwent endoscopic laser resection to highlight the urethra and cause severe end hematuria permanently.

**Case presentation:**

In 2019, a 37-year-old woman came to the hospital because of hematuria and a dark red extraurethral mass. CTU inspection showed: filling defect between the right ureter and the bladder at the entrance of the bladder. After ureteroscopy, it was found that the ureteral mass came out of the urethral orifice. Then, under the direct view of the ureteroscope, a Ho:YAG laser was used to remove the tumor by cutting off along the its base, and the patient was discharged 3 days after the operation.

**Conclusion:**

Urethral polyps from the ureter should be considered in the differential diagnosis of urethral neoplasms. Ho:YAG laser resection under ureteroscopy is an effective option for treating UFP, but be careful of ureteral stricture after surgery.

## Background

Ureteral fibro-epithelial polyp (UFP) is a rare benign ureteral tumor that causes symptoms, such as hematuria, low back pain, frequent urination, and difficulty in urinating. Various factors, including congenital malformation, obstruction, trauma, irritation, infection, and specific-derived hormone imbalance, contribute to this disease [1]. Its specific etiological mechanism is still unknown. Ludwig reported that the median size of UFP was 4 cm, and no statistical difference was observed for the occurrence on both sides [2]. Moreover, the upper part of the ureter was the most common origin of these polyps. In this case, we report a severe lower end ureteral polyp in middle-aged women with severe terminal hematuria and permanent protrusion of the urethra from the polyp. Reports of such a large UFP outside the urethra are rare.

## Case presentation

**Fig. 1:**
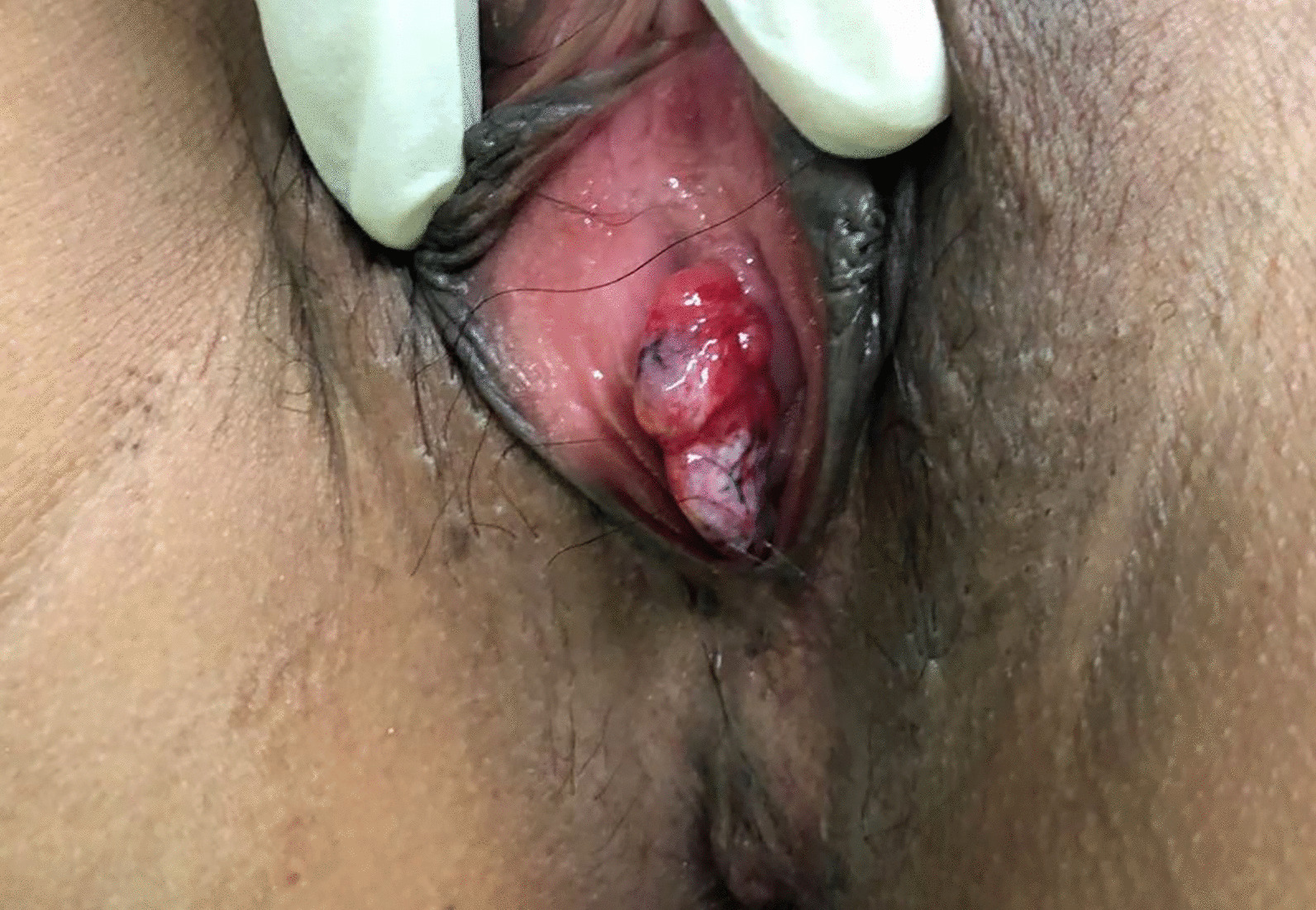
Long mass protruding from the urethral opening

**Fig. 2:**
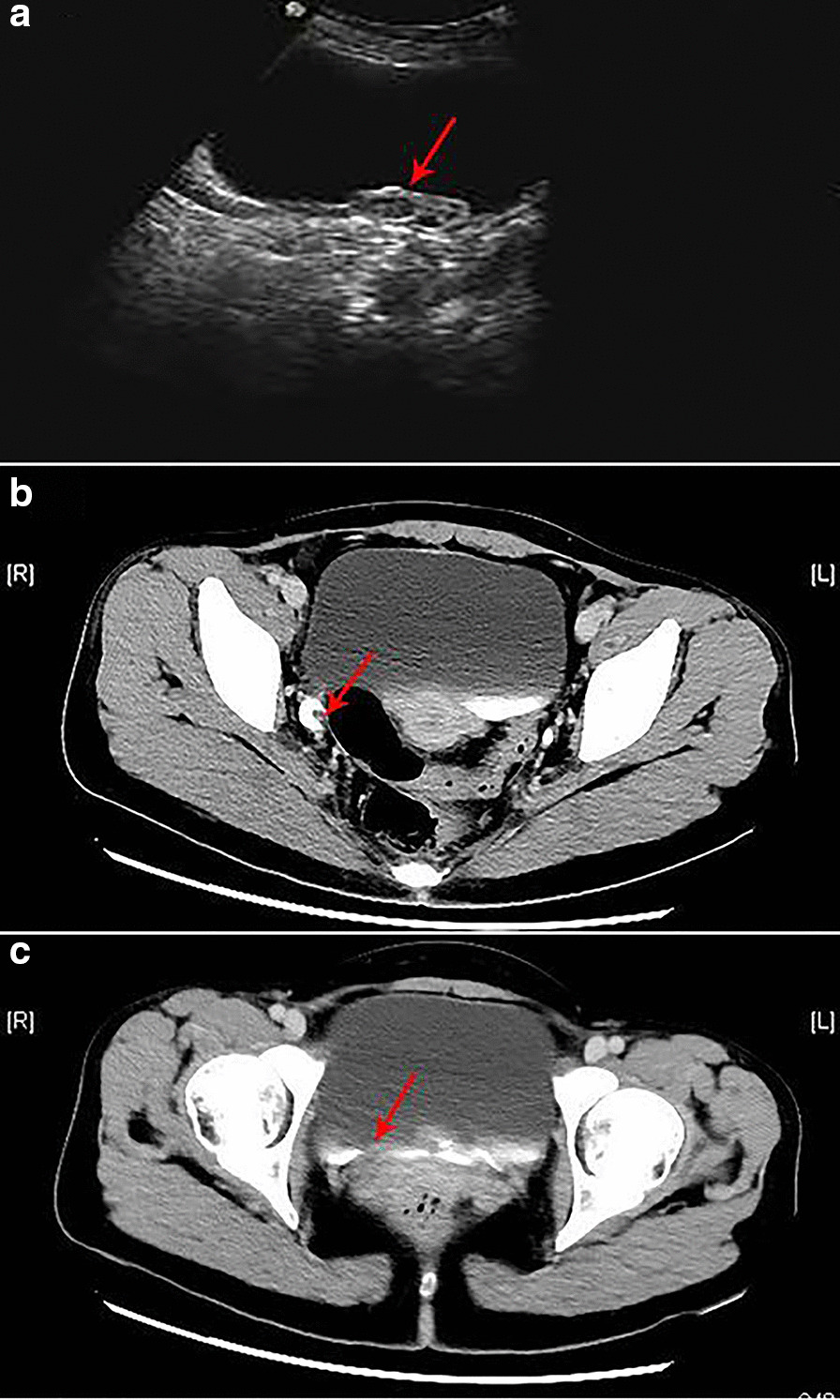
**a** First-class echo at the bladder opening in the lower ureter. **b** Filling defect between the right ureter and the bladder at the entrance of the bladder. **c** Filling of the bladder is acceptable, and no nodular filling defect is observed in the posterior wall. (shown by the red arrow)

**Fig. 3:**
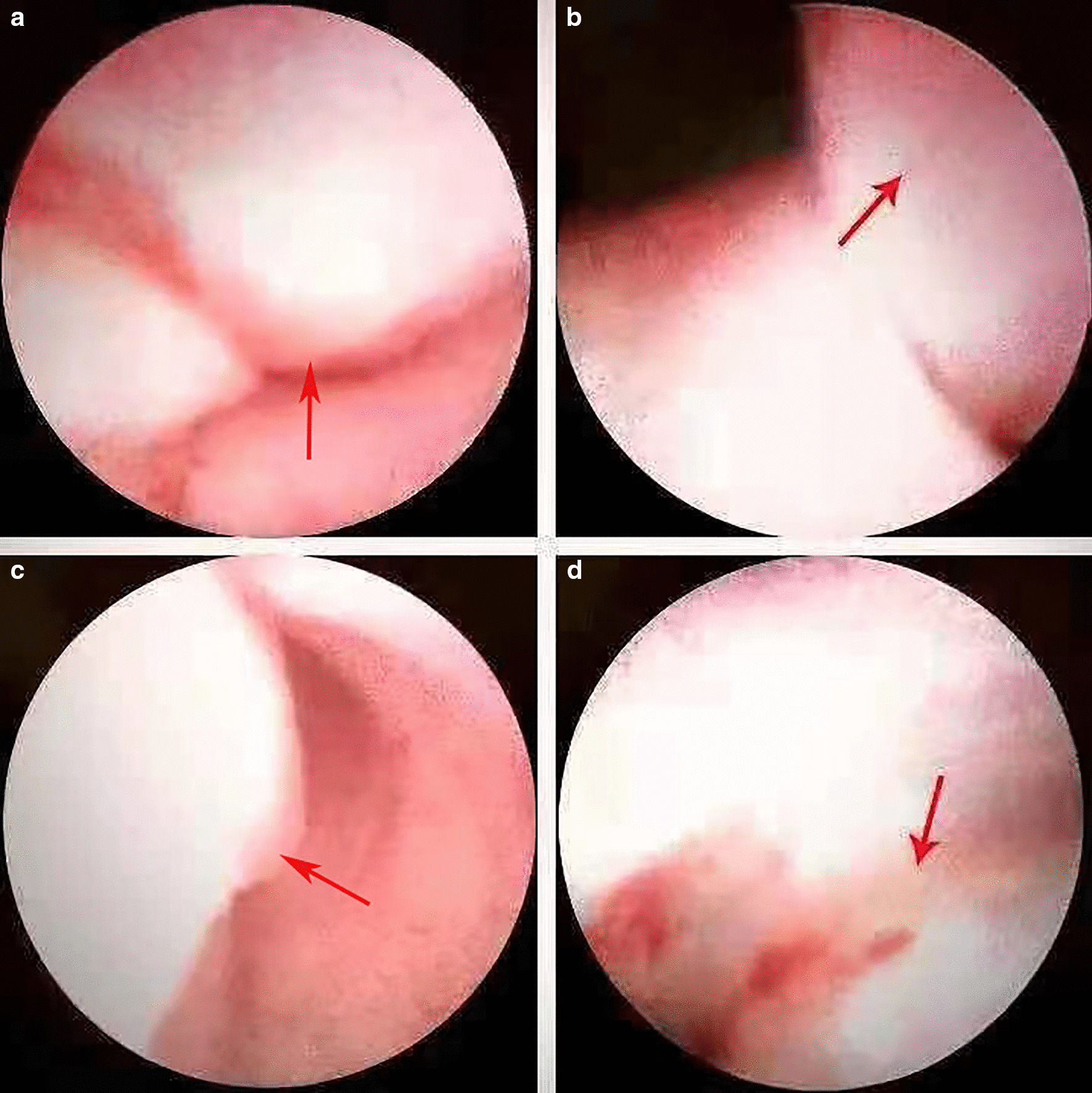
Ureteroscopy during surgery. **a** Sees the tumor protruding through the urethra; **b** sees the tumor protruding from the opening of the right ureter; **c** does not completely block the ureteral cavity; **d** sees the base of the tumor (shown by the red arrow)

**Fig. 4:**
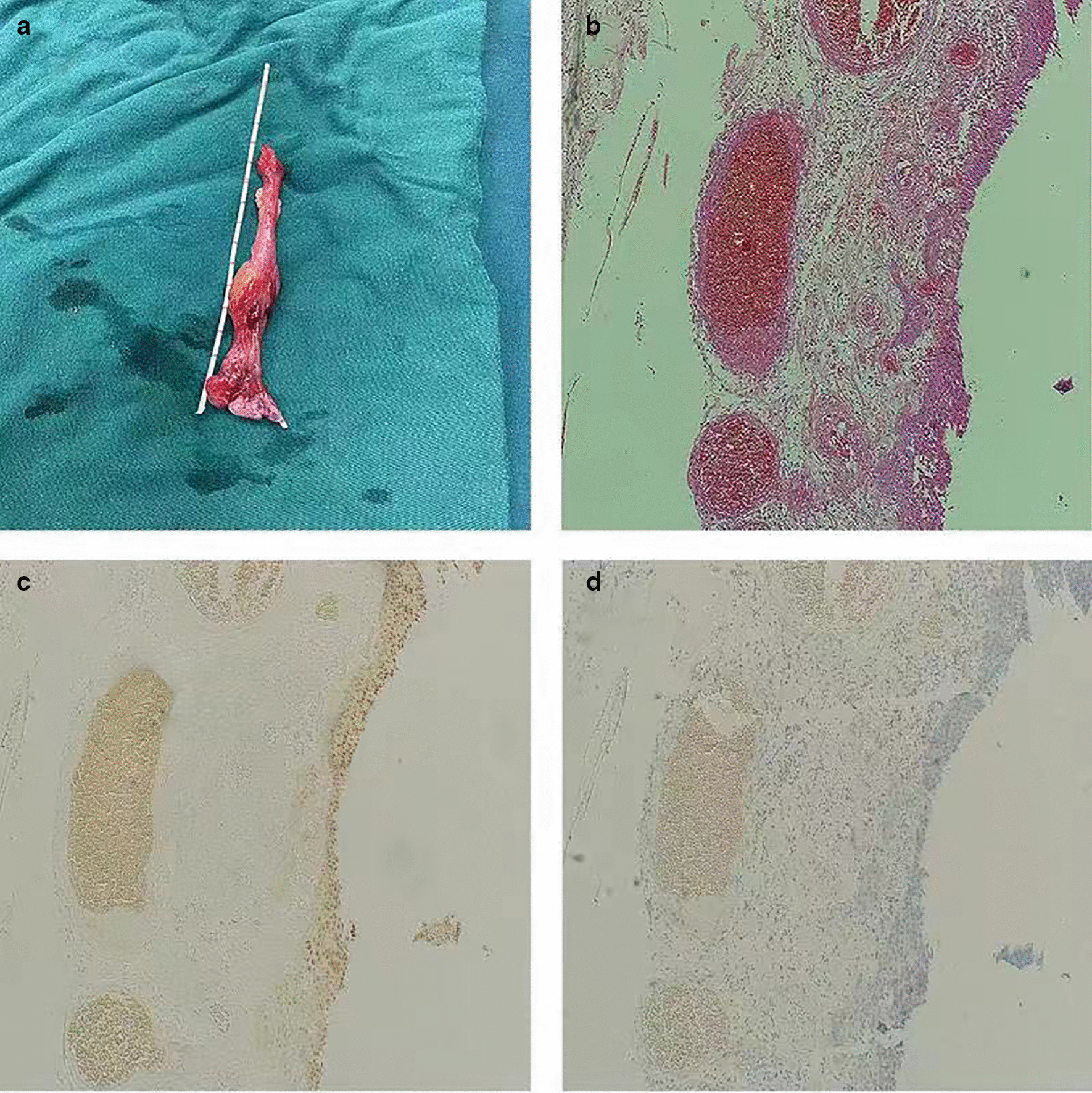
**a** Gross specimens and pathology after surgery. The total length of the tumor is approximately 12 cm, the surface is smooth, the diameter of the tube is approximately 0.8–1.8 cm, the cut surface is grayish red, and the edema is translucent. **b** H & E staining (× 100) showed that urothelial hyperplasia conformed to polypoid changes, but no dysplasia or malignancy. **c** Immunohistochemistry indicates positive Gata3 nucleus. **d** Immunohistochemistry indicates negative CK20 cytoplasm

A 37-year-old female patient experienced intermittent hematuria with dysuria for 10 days, with dark red vegetation prolapsed from the urethra during urination. The patient had no evident urinary urgency, pyuria, waist discomfort, or fever. During the physical examination, a dark red strip-shaped mass protruded from the urethral orifice. This mass was soft, and its root could not be explored (Fig. 1). Ultrasound showed a 36 mm × 17 mm mild hydronephrosis in the right kidney, and other echoes were observed at the opening of the bladder in the lower part of the right ureter (Fig. 2a). The inner diameter of the lower end of the right ureter was 8 mm, and the inner diameter of the upper segment was 6 mm. Computed tomography urography (CTU) examination showed a strip filling defect at the entrance of the bladder on the right ureter (Fig. 2b). The filling of the bladder was satisfactory, and a nodular filling defect was observed on the posterior wall (Fig. 2c). Afterwards, the patient received ureteroscopy diagnosis under general anesthesia. During operation, the exposed mass was held with tissue forceps, and the tension was slightly maintained. A ureteroscope was used to examine the bladder through the urethra (Fig. 3a), and the mass was seen protruding from the right ureteral opening (Fig. 3b). The silk and ureteroscope were utilized to assess the right ureter, and the base of the mass was found in the middle and lower section of the right ureter (Fig. 3d). The mass was strip-shaped, the surface was smooth, and no obvious hemorrhage and necrosis were observed. The basal mucosa of the mass showed edema, and the tumor tissue did not completely block the ureteral cavity (Fig. 3c). Then, under the direct view of the ureteroscope, a 200 mm Ho:YAG laser fiber with an energy setting of 1 J and a frequency of 15 Hz was used to remove the tumor by cutting off along the its base. The tissue was clamped out of the tumor, and the right ureter was placed with an F5 double J tube. The long grape-shaped mass was 12 cm in length with a smooth surface. The diameter of the tube was approximately 0.8–1.8 cm, and the edema was translucent (Fig. 4a). Combined with hematoxylin and eosin staining (Fig. 4b) and immunohistochemical nucleus Gata3 (+) (Fig. 4c), CK20 (−) in the cytoplasm (Fig. 4d) was diagnosed as a polyp formed by ureteric epithelial proliferation. The double J tube was removed 3 months after the operation by ureteroscopy which was performed to review the wound. The patient received the urinary system B-ultrasound 6 months after the operation, indicating no ureteral stenosis or hydronephrosis.

## Discussion and conclusion

UFPs are rare benign ureteral tumors, accounting for 1% of all urogenital tumors. The proximal ureter is the most common origin of these polyps [3], and polyps from the lower urinary tract are uncommon. The symptoms of UFP depend on the size and condition of the polyp. The typical symptoms include hematuria, low back pain, frequent urination, and dysuria [2]. Although this patient has hematuria and mild hydronephrosis, but she came to the hospital due to an unknown mass protruding from the urethra.

Imaging studies can help diagnose UFP. Intravenous pyelography or CTU can show filling defects and can also reveal the possibility of hydronephrosis, which suggests the patient’s renal function status. Most importantly, distinguishing UFP from transitional cell carcinoma based on imaging findings alone may be difficult [4]. Thus, ureteroscopy is also needed for patients suspected of UFP assessed by intravenous pyelography, computed tomography or retrograde urography for diagnosis and treatment. The shape, number of tumors, the base diameter, and the location of the obstruction can be determined simultaneously by ureteroscopy. Preliminary judgments about benign and malignant tumors will be noted.

To date, no uniform standard is available for the main treatment methods for ureteral polyps, and surgical removal of polyps is still the preferred solution. In this case, ureteroscopy reveals that the single polyp is extremely long and originated from the lower part of the ureter. The strip-shaped mass has a smooth surface with no obvious hemorrhage and necrosis. The mucosa at the base of the mass is edema. For ureteral polyps, the surgical field of the basal floor is clear, and then intraluminal resection is selected. At the same time, a Double-J stent is placed to prevent ureteral stricture after surgery. Endoscopic resection has minimal trauma to the patient but with rapid post-operative recovery and minimizes the risk of ureteral stenosis and renal impairment [5]. For patients with malignant and multiple tumors, poor ureteroscopy is unable to expose the base of the polyp, which is not suitable for endoscopic resection. In this case, laparoscopic or open surgery can be opted.

We have reported a case of a huge UFP originating from a 12 cm UFP distal to the right ureter, which completely protrudes from the urethral opening, and the polyps were removed by laser under ureteroscopy. In 2013, Ali Momenzadeh et al. reported a case of large UFP presenting a vegetative bladder mass excised ureteroscopically and recommended further assessment for any mass juxtaposed vesicoureteral junction and also suggest ureteroscopically approach to distal UFPs, it was the first report from IRAN [6]. At present, the literature has reported many similar cases of ureteral polyps extending into the bladder cavity, only one case report a UFPs with permanent extension beyond the bladder cavity through the urethra, this 19-year-old patient had two UFPs from the distal left ureter, they used cautery to remove the entire polyp under the endoscope [7]. According to the Ludwig D.J, due to advances in technology, more UFP cases had been treated by electrocautery or YAG laser through ureteroscopy since 1980s, with no reported recurrence. Abdominal CTU after 3 months and urinary system ultrasound after 1 year to detect possible recurrence (ureteral stenosis and hydronephrosis) were recommended during the surveillance of this pathology [2]. Our case reports a huge UFP with permanent extension beyond the bladder cavity through the urethra, which is the first case in China. Simultaneously, immunohistochemistry assay was performed to strong evidence for our diagnosis.

## Data Availability

Not applicable.

## Abbreviations

UFP: Ureteral fibro-epithelial polyp
CTU: Computed tomography urography
